# Supplementary material for: NRPS-like ATRR in Plant-Parasitic Nematodes Involved in Glycine Betaine Metabolism to Promote Parasitism
Source: Int J Mol Sci. 2024 Apr 12;25(8):4275. doi: 10.3390/ijms25084275 (PMC11050029; doi:10.3390/ijms25084275)
Supplement: Supplementary file 1 [file ijms-25-04275-s001.zip › Supporting Information.pdf]

Supporting Information

Suppl. Table S1. Summary of NRPS related gene clusters and genomes analyzed in this study.

Suppl. Table S2. List of life-stage specific transcriptomes of *M. incognita* used for MiATRR CDS matches in this study.

Suppl. Table S3. Results of statistical analysis in this study.

Suppl. Table S4. List of primers used in this study.

Suppl. Figure S1. 3D structural alignment of MiATRR and *A. nidulans* ATRR. A, A-domain; B, R_1 domain; C, R2 domain. Pink: MiATRR, green: *A. nidulans* ATRR.

Suppl. Figure S2. Confirmation of *Arabidopsis* positive transformants by PCR amplification of the target gene *Miatrr*, with plant gene UBP22 as control.

Supple. Figure S3. Effects of glycine betaine and choline on *M. incognica* survival and *Miatrr* expression after free-living J2s exposed to different concentration of the chemicals for 72 h. Percentage of *M. incognica* survival under stress of glycine betaine (A) and choline (B). Relative expression levels of *Miatrr* under stress of glycine betaine (C) and choline (D).
