# Supplementary figures and images for: NRPS-like ATRR in Plant-Parasitic Nematodes Involved in Glycine Betaine Metabolism to Promote Parasitism
Source: Int J Mol Sci. 2024 Apr 12;25(8):4275. doi: 10.3390/ijms25084275 (PMC11050029; doi:10.3390/ijms25084275)

A domain align

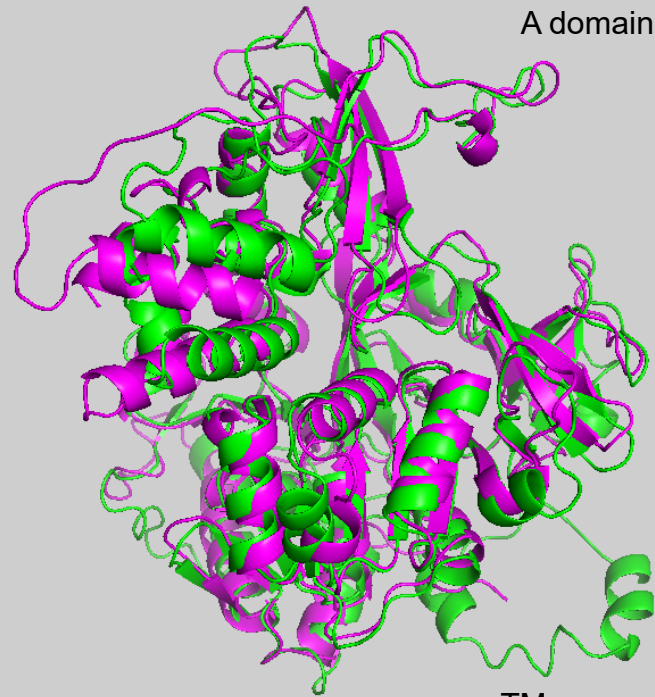

TM-score > 0.82

R1 domain align

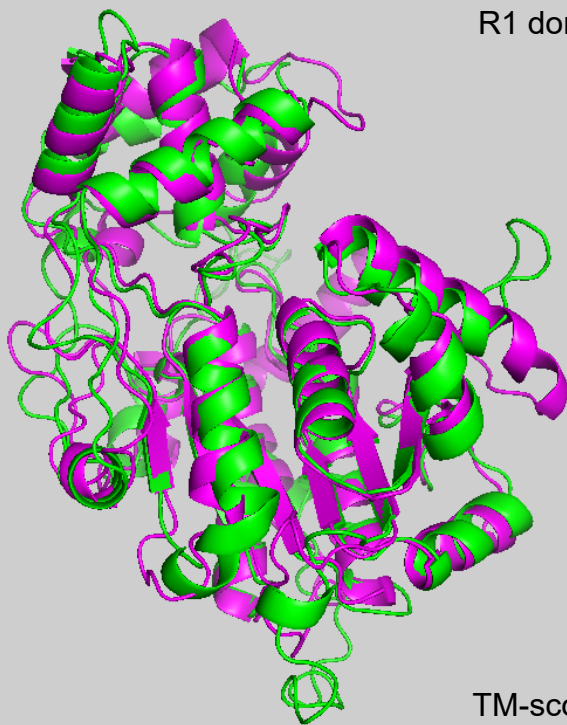

TM-score > 0.82

R2 domain align

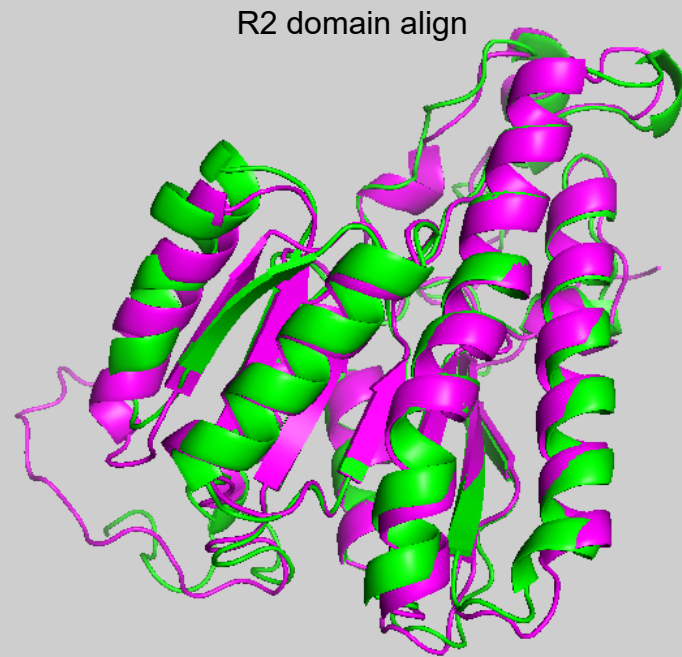

TM-score > 0.88

Supplement: Supplementary file 1 [file ijms-25-04275-s001.zip › Supplementary Figure S1.pdf]

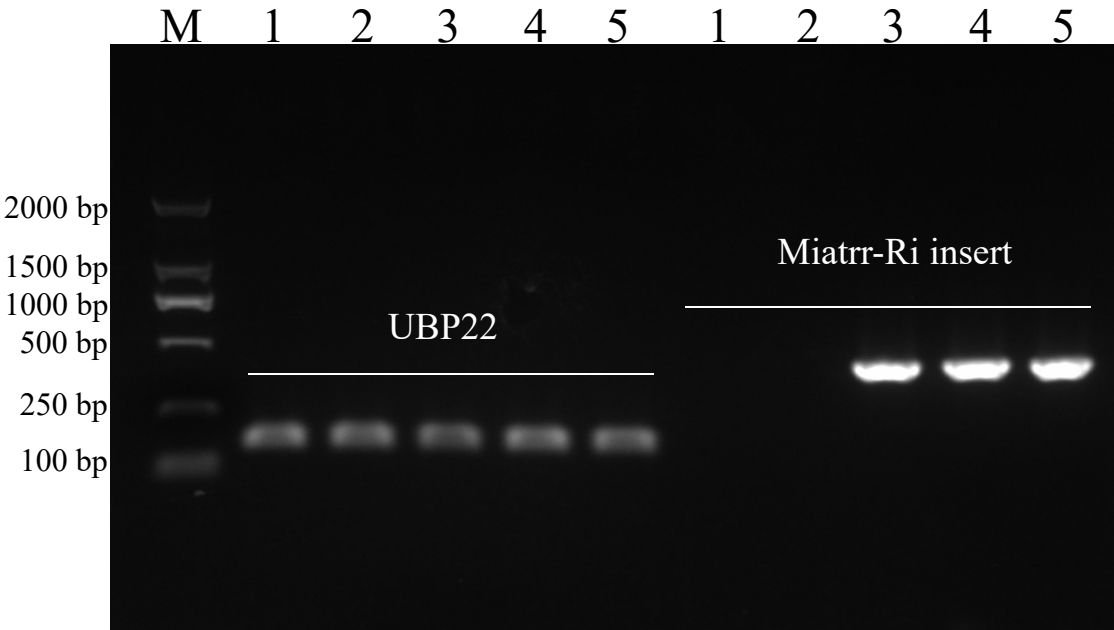

Supplement: Supplementary file 1 [file ijms-25-04275-s001.zip › Supplementary Figure S2.pdf]

**A**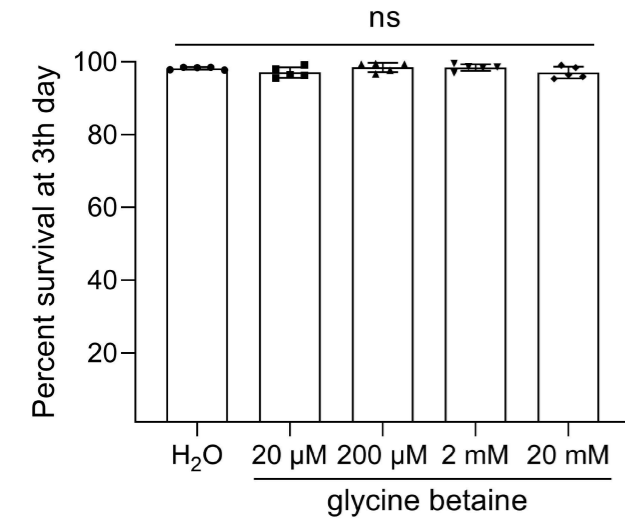**B**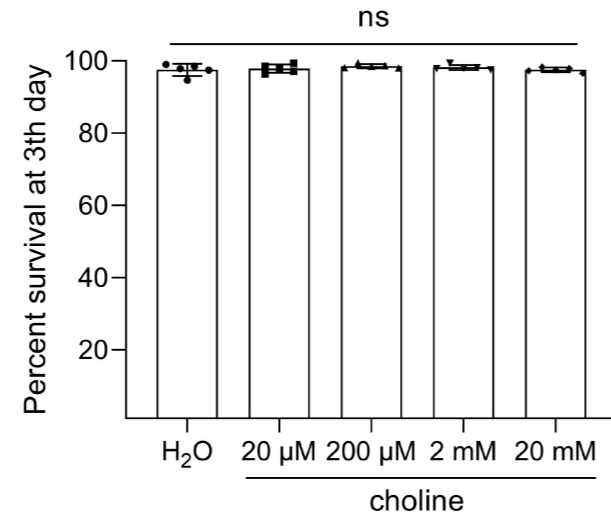**C**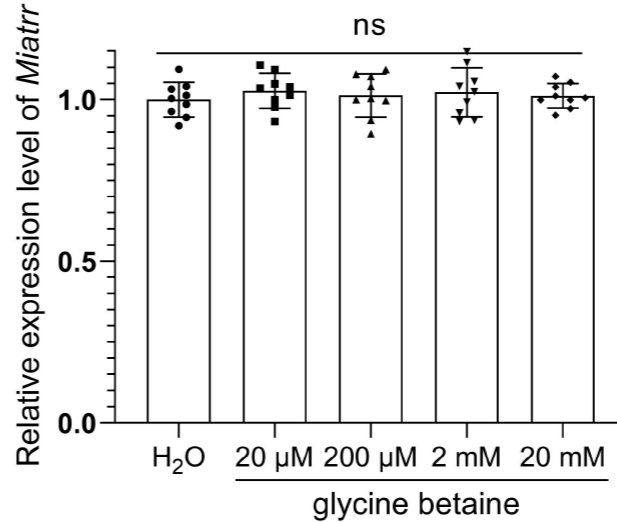**D**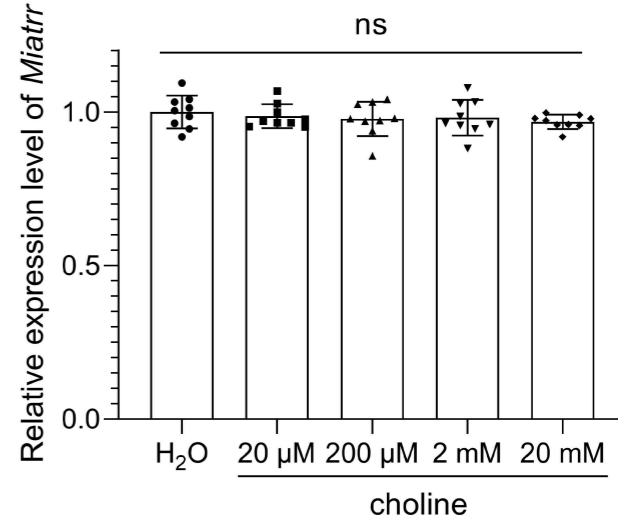

Supplement: Supplementary file 1 [file ijms-25-04275-s001.zip › Supplementary Figure S3.pdf]
